# Dek Loss Induces Sex-Dependent, Task-Specific Cognitive Deficits and Reprograms the Hippocampal Transcriptome in Mice

**DOI:** 10.21203/rs.3.rs-7577113/v1

**Published:** 2025-10-17

**Authors:** Kaitlyn Gardner, Taylor E. Lange, Marla K. Perna, Susanne I. Wells, Michael T. Williams, Charles V. Vorhees, Matia B. Solomon, Lisa M. Privette Vinnedge

**Affiliations:** Cincinnati Children’s Hospital Medical Center; University of Cincinnati College of Medicine; Cincinnati Children’s Hospital Medical Center; Cincinnati Children’s Hospital Medical Center; Cincinnati Children’s Hospital Medical Center; Cincinnati Children’s Hospital Medical Center; University of Cincinnati College of Arts and Sciences; Cincinnati Children’s Hospital Medical Center

**Keywords:** DEK, cognitive dysfunction, sex differences, memory, hippocampus

## Abstract

Cognitive decline with aging, and some neurodegenerative conditions like Alzheimer’s disease, disproportionately affects females yet few mechanisms beyond steroid hormone signaling fully explain this sex-specific vulnerability. The chromatin-remodeling DEK protein, upregulated by estrogen and progesterone and broadly expressed in the brain, including the hippocampus, may be one such mechanism. We have previously linked DEK loss with indices of neuronal dysfunction, including increased DNA damage, impaired neurite development, and apoptosis, suggesting a potential neuroprotective role. Here, we investigated the molecular and behavioral consequences of *Dek* loss in vivo. Female *Dek* constitutive knockout (cKO) mice exhibited a sex-specific behavioral phenotype, with impairments in sensorimotor gating, as measured by pre-pulse inhibition, and in reversal learning in the Morris Water Maze. These findings are suggestive of deficits in pre-attentive sensory processing and cognitive flexibility, respectively. Notably, these cognitive deficits were not observed in male *Dek* cKO mice and were not attributable to differences in general learning ability, locomotor activity, or anxiety-like behavior. The absence of impairment in object recognition and conditioned fear learning and memory in females suggests that the effects of DEK loss are task-specific and likely brain region-specific. Transcriptomic analysis of hippocampal tissue revealed differentially expressed genes related to inflammation, metabolism, and neuropeptide signaling in all *Dek*-cKO mice, along with a distinct female-specific transcriptomic profile indicative of impaired neuronal function. Combined, we report for the first time that DEK supports certain aspects of cognitive function, particularly in females. These data may be relevant for understanding sex differences in some cognitive disorders.

## Introduction

Clinically impaired learning and memory can arise from congenital conditions, traumatic brain injuries, aging-associated cognitive decline, and neurodegenerative conditions like Alzheimer’s disease (AD). The hippocampus is responsible for spatial, long-term, and episodic memory and, as the site of persistent neurogenesis, hippocampal dysfunction and atrophy are hallmarks of pathological conditions that impact cognition and memory. The hippocampus may also be a site for sex differences in certain forms of learning and memory, as both androgen and estrogen receptors are expressed in this region.[1] Among neurodegenerative conditions that impact cognitive function, there are known sex differences in their pathogenesis. Men are more likely to experience cognitive impairment with Parkinson’s disease while women are disproportionately more likely to be diagnosed with AD and age-related dementias.[2, 3] The female-biased prevalence in some age-related dementias, like AD is not solely due to the longer life-expectancy of women.[4, 5] One hypothesis is that age-associated loss of neuroprotective estrogens either in the gonads, brain, or both, contributes to this female-biased increase in dementia. For example, menopause is associated with a decline in verbal memory and early menopause is associated with an increased risk for AD.[6] However, the potential role of non-hormonal factors that may also contribute to sex differences in cognitive performance, age-associated cognitive decline, and dementias remains poorly understood. One factor that may contribute to sex-specific differences in cognitive function is the DEK protein. *DEK*, an estrogen receptor alpha (ER-α) target gene, is a histone chaperone essential for DNA replication, genome stability, chromatin organization, and transcriptional regulation.[7–9] *DEK* is expressed in almost all tissues including the brain, but primarily in proliferating progenitor cells.[10, 11] The majority of previous reports have focused on the role of *DEK* as an oncogene, particularly in solid tumors, where it is commonly overexpressed.[12–18] Our group and others have shown that *DEK* loss in cancer cell lines leads to impaired activation of DNA damage repair pathways,[19, 20] cellular senescence,[21] and cell death.[22–25] In addition, using SH-Sy5y cells differentiated to acquire a neuronal phenotype, we demonstrated that *DEK* knockdown correlated with cellular and molecular features associated with AD, including cell death and increased pathogenic Tau accumulation. [26] These findings are supported by complementary in vitro [27] and in vivo findings [26–28]. We were the first to report that *DEK* is expressed in multiple brain regions critical for learning and memory, such as the hippocampus and medial prefrontal cortex, and that it is likely present in various cell types including astrocytes, neurons, and microglia in both the mouse and human brain.[11, 29] Furthermore, we reported sex-specific differences in DEK protein expression in the murine brain, including the finding that females have higher DEK expression in the CA1 region of the hippocampus compared to males[11], which is a critical area for spatial learning and memory and episodic-like based memory in rodents.[30, 31] Using human postmortem brain tissue, we found that older women with schizophrenia-associated dementia show decreased DEK protein expression in the anterior cingulate cortex, a region important for integrating emotional and cognitive information, compared with sex- and age-matched healthy controls. [32] This decrease was inversely correlated with dementia severity, suggesting that lower DEK levels track with more severe cognitive decline. Notably, this association was not observed in elderly men with schizophrenia-associated dementia, pointing to a female-biased link between decreased DEK expression and dementia. Using transcriptomic data, we also showed that *DEK* levels are highest in the fetal human brain and decrease with age.[29, 33] Additionally, *DEK* was identified as a differentially expressed gene in Huntington’s disease [33], a neurodegenerative disorder characterized by progressive cognitive decline.[34] This finding suggests that DEK may play a role in brain disorders beyond schizophrenia-associated dementia and highlights the need to further explore its function in cognitive health. Combined, our prior work and that of other groups suggest that DEK loss is associated with molecular and cellular correlates of cognitive dysfunction, This study aims to bridge the gap between DEK protein expression data from murine and human brains by examining the functional and molecular consequences of *Dek* loss on memory in adult female and male *Dek* knockout (KO) mice compared with wild-type (WT) control mice. Based on the link between decreased DEK expression to AD phenotypes in human cell models and the selective decrease of DEK in elderly women living with dementia, we hypothesized that female *Dek* KO mice would exhibit behaviors indicative of cognitive dysfunction. To examine the molecular consequences of *Dek* loss in the murine brain via bulk RNA-Seq, we chose to focus on the hippocampus because it is one of the brain regions with highest *Dek* expression, it is essential for memory, and it is impacted by memory impairment in neurodegenerative disorders like AD. Here, we show that *Dek* loss results in some cognitive deficits and a unique transcriptomic signature that indicates neuroinflammation, and alterations in synaptic function and glutamate/calcium homeostasis. This includes changes to genes associated with neuron vitality and function and links these changes to epigenetic mechanisms via histone post-translational modifications, a well-established function of DEK.

## Results

### Impact of Dek Loss in Males and Females on Behavior.

**Figure 1.**
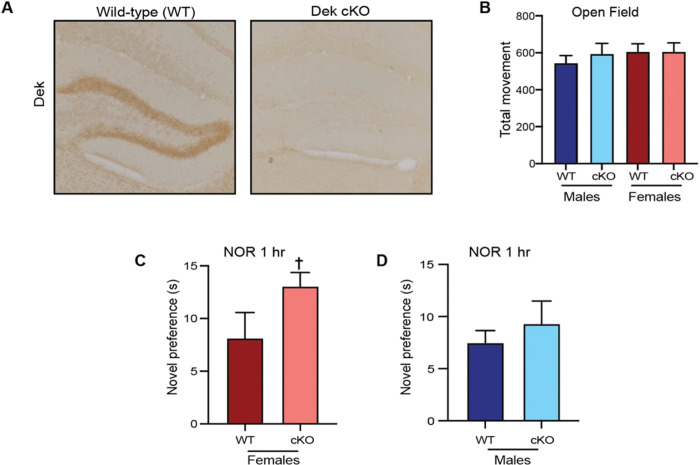
Loss of DEK expression in the brain causes sex-specific memory impairment using the novel object recognition test in adult mice. (A) Immunohistochemical staining for DEK reveals prominent staining in the dentate gyrus in wild-type mouse brain while expression is absent in brain tissue from *Dek* conditional knockout (*Dek* cKO) mice. (B) All mice, regardless of sex or genotype, performed similarly in the open field test for locomotor activity. (C-D). The novel object recognition (NOR) task, at the 1 h retention interval for females, there was a trend of genotype (t(16.689) = 1.77, p = 0.0949) (C), but no difference in males (panel D). █p<0.1 All mice were adults; N=12 WT females, 10 *Dek* cKO females, 14 WT males, 7 *Dek* cKO males.

To investigate the effect of *Dek* loss on behavior, *Dek* cKO mice were generated by breeding the *Dek*^*fl/f*l^ mice with CMV-Cre mice to generate a constitutive knockout (cKO) as reported.[35] Loss of DEK expression was verified via immunohistochemistry staining in the dentate gyrus in WT versus the *Dek* cKO mice (Fig. 1A). Given previous findings that diminished DEK expression significantly impacts neurite formation and neural viability *in vitro*, we investigated the consequences of *Dek* loss on behavior. For all studies, we analyzed for the effects of genotype by sex. Open-field activity was assessed for 1 h to ensure that there was no change in exploration or habituation activity of the mice due to *Dek* loss. There were no significant locomotor changes in *Dek* cKO male mice (Mean (beam interruptions) ± SEM: cKO: 594.9 ± 50.0 vs. WT: 545.2 ± 39.6) or female mice (cKO: 606.4 ± 46.9 vs. WT: 606.1 ± 42.8) compared with WT mice (Fig. 1B). *Dek* cKO mice also did not display any difference in preference for central vs. peripheral movement, suggesting they did not have differences in anxiety-like behavior.

The novel object recognition (NOR) test was used to determine whether the loss of *Dek* expression affected recognition memory. Following familiarization, the 1 h retention showed no differences in *Dek* cKO in time spent investigating the novel object compared with WT mice (t(16.689) = 1.77, p = 0.0949 for females; t(9.618) = 0.74, p = 0.48 for males, Fig. 1C). There also were no differences were observed at the 24 h retention interval (not shown). For males, there was no genotype difference at the 1 (Fig. 1D) or 24 h (not shown) retention intervals.

To explore the effects of *Dek* cKO loss on spatial learning, reference memory, and cognitive flexibility, the Morris water maze (MWM) was used. Before assessing spatial learning, mice were given cued training in the maze from a fixed start to a fixed and visible platform. For males there were no significant differences on training trials (**Fig S1A**). For females there was a genotype × trial trend (F(5,69.4) = 1.99, P = 0.0910; **Fig S1A**) which was primarily seen on trials 1, 4, and 5. Next, mice were tested for spatial learning to find a hidden platform with different start positions on each trial with 4 trials/day for 5 days. For males there were no differences in latency (**Fig. S1B**), speed (**Fig. S1C**) distance, or path efficiency (not shown). In females there was a significant interaction of genotype × day for latency (F(4,52.7) = 2.87, p = 0.0319, **Fig S1B**). Further analyses showed this effect was confined to a trend on day 5 wherein *Dek* cKO females had longer latencies than WT females (p’s = 0.10). There were no differences in females for distance or path efficiency (not shown). There was a trend for a genotype × day interaction for swimming speed in females (F(4,52.1) = 2.39, p = 0.0617) with the *Dek* cKO females swimming slightly slower on day 5 (**Fig S1C**, mean speed shown). There was an effect of genotype × day for swimming speed (F(4,58.8) = 2.96, p = 0.0268), but further analyses did not show any differences between genotypes (**Fig S1C** and not shown). 24 h after the last acquisition trial, mice were given a probe trial with the platform removed as a test of reference memory. For the males, there was a trend for the *Dek* cKO males to be further from the platform site than the WT males (t(19) = 1.85, p = 0.0793, **Fig S1D** top) but no differences in site entries (**Fig S1E** top).There were no differences between the females for average distance from the platform site or site entries (**Fig S1D and S1E**, bottom).

**Figure 2.**
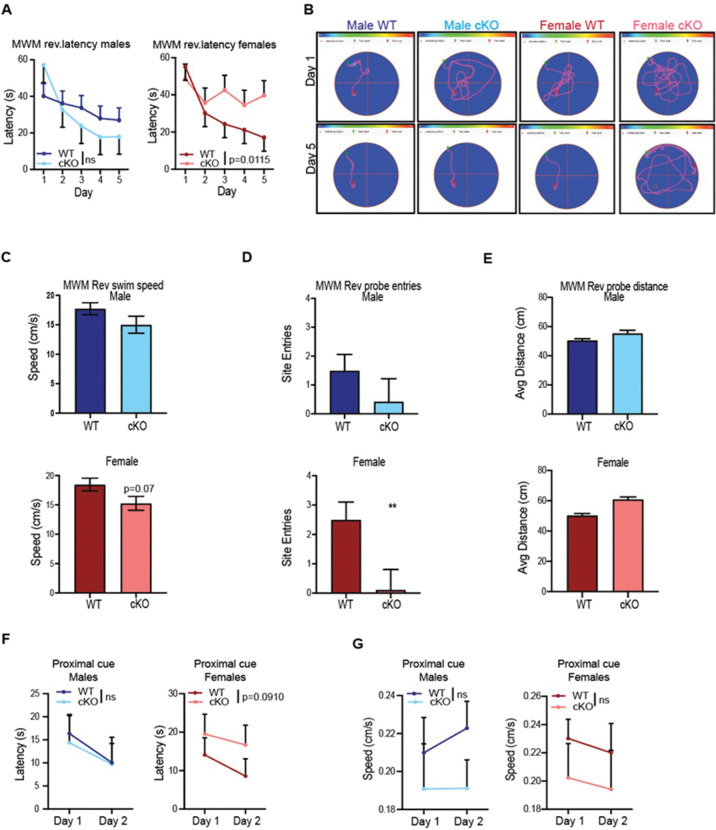
*Dek* loss causes impaired spatial memory in females, but not males. There were no differences in performance during cued trials in the Morris water maze on either latency (A) or speed (B). On hidden platform acquisition trials for females, there was a genotype × day interaction for latency (F(4,52.7) = 2.87, p = 0.0319). On the probe trial 24 h after the last acquisition trial, there were no effects on entries for females and males. For average distance to the former platform site there was no effect in females, but there was a trend in cKO males to be further away (t(19) = 1.85, p = 0.0793).. On reversal trials for females for latency there was a genotype × day interaction (F(4,61.2) = 3.54, p = 0.0115; female cKO mice had longer latencies than WT females on day 5 with a trend on day 3 (panel C). There were no differences in males. Example path tracings are shown in panel D. On the reversal probe trial for females, there was an effect of genotype on platform zone entries (t(11.384) = −2.82, p = 0.0161), but no effect in males. For females and males there were no differences in average distance from the former platform location. Group sizes: N=12 WT females, 10 *Dek* cKO females, 14 WT males, 7 *Dek* cKO males.

On MWM reversal, for males, there were no differences on any of the measures (Fig. 2A-C). For females there was a genotype × day effect on latency (F(4,61.2) = 3.54, p = 0.0115) (Fig. 2A). Further analyses of each day showed a trend on day-3 (p = 0.096) and a significant effect on day-5 (p = 0.0220). For path efficiency there was a genotype × day effect (F(4,61.5) = 2.57, p = 0.0469) in which *Dek* cKO females exhibited less efficient paths to the goal than WT females on day 5 (p = 0.0243) (Fig. 2B). On swimming speed, there was a genotype trend (F(1,20.3) = 3.46, p = 0.0776) in which WT females swam faster than *Dek* cKO females (WT: 18.48 cm/s vs cKO: 15.28 cm/s:, Fig. 2C). For distance there was a genotype × day trend (F(4,61.6) = 2.49, p = 0.0525, not shown). On the reversal probe trial there was a significant genotype effect in females (t(11.384) = −2.82, p < 0.0161) in which *Dek* cKO females had fewer site entries than WT females (Fig. 2D). There were no differences between females for average distance to the platform site; swimming speed showed a genotype trend (t(19) = −1.99, p < 0.0609, Fig. 2E). For males, there were no differences on probe trial entries or distance (Fig. 2D-E). A day after the reversal probe trial, mice were given cued/visible platform trials as a test of proximal cue learning for 4 trials/day for 2 days with curtains closed around the pool, a marked platform and with both start and platform positions changed on every trial. There were no genotype effects on these trials for males or females (Fig. 2F,G).

**Figure 3.**
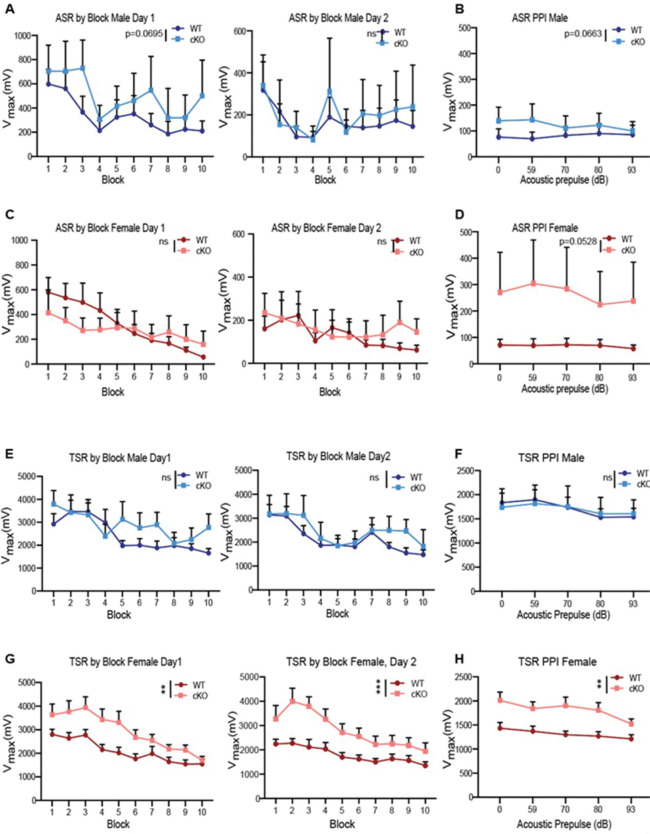
*Dek* loss results in sex-specific impairment in tactile startle response but not acoustic startle responses. (A,C) There is no difference in acoustic startle V_max_. Acoustic stimulus is a 20 ms, 120 dB (SPL) mixed frequency sound burst with 1.5 ms rise time. (B,D) There are no statistically significant differences between *Dek* cKO and WT mice for V_max_ on acoustic prepulse inhibition trials. Of note, there is a trend for increased V_max_ in Dek cKO males and females compared with WT males and females. (E,G) Tactile startle showed no differences between WT and *Dek* cKO males on either day 1 or day 2. However, there were more pronounced and consistent increases in startle in female *Dek* cKO mice compared with WT females, differences supported by an effect of genotype (F(1,22.7) = 10.48, p = 0.0037) in which the cKO females were hyperreactive. On Day 2, females again had a significant genotype effect (F(1,21.4) = 17.19, p = 0.0004) (panel G, right). The tactile stimulus was a 20 ms 60 psi air puff, delivered to the dorsal surface of the mouse through a tube inserted in the top of the animal holder. (F, H) There were no differences between WT and *Dek* cKO males for tactile PPI. Compared with WT females, female *Dek* cKO showed reduced prepulse induced suppression (panel (H): F(1, 20.6) = 10.69, p =0.0037) that was not observed in males (panel F). N=12 WT females, 10 *Dek* cKO females, 14 WT males, 7 *Dek* cKO males

*Dek* cKO mice were then assessed for acoustic and tactile startle responses. For acoustic startle response (ASR) in males there was no effect of genotype but there was a genotype × trial block trend (F(9,141) = 1.82, p = 0.0695) on Day 1 (Fig. 3A). Males demonstrated no differences in tactile startle response (TSR, Fig. 3E) there were no effects between males. There were also no effects for females for ASR (Fig. 3C). Tactile startle produced significant differences between cKO and WT female mice on Day 1 (F(1,22.7) = 10.48, p = 0.0037) and Day-2 of testing (F(1,21.4) = 17.19, p = 0.0004) (Fig. 3G) with female *Dek* cKO mice hyperreactive compared with WT females.

On Day 3, mice were given prepulse inhibition trials (PPI) with an acoustic prepulse and an acoustic or tactile pulse. For acoustic PPI in males, there was a genotype × prepulse trend (F(4,55.8) = 2.34, p = 0.0663) (Fig. 3B). There were no effects in males on PPI trials with a tactile pulse (Fig. 3F). For acoustic PPI in females there was no genotype effect but there was a genotype × prepulse interaction (F(4,54) = 2.50, p = 0.0528 (Fig. 3D). Further analyses at each prepulse level failed to identify a particular prepulse level associated with this interaction. On trials with a tactile pulse, for females there was a genotype main effect (F(1,20.6) = 10.69, p = 0.0037) (Fig. 3H). In both cases of PPI testing, *Dek* cKO females were hyperreactive compared with WT females.

Conditioned freezing showed no differences in females or males on Day-1 habituation (**Fig S2A**). On Day-2 (conditioning) there was no effect among females, but there was a genotype (F(1,19) = 7.78, p = 0.0117) and genotype × interval effect among males (F(1,19) = 6.33, p = 0.021). For the interaction for males there was no effect on Interval-1 (habituation), but there was an effect on interval-2 (conditioning) in which *Dek* cKO males showed less freezing in response to the stimuli with foot-shock than WT males (WT: 32.85 ± 1.22 vs. *Dek* cKO: 26.95 ± 1.72) (**Fig S2B**). On Day-3 (contextual memory) and Day-4 (cued memory plus extinction) there were no genotype effects in either sex (**Fig S2C-H**).

### Sex Differences in the Transcriptomic Consequences of Dek Loss

**Figure 4.**
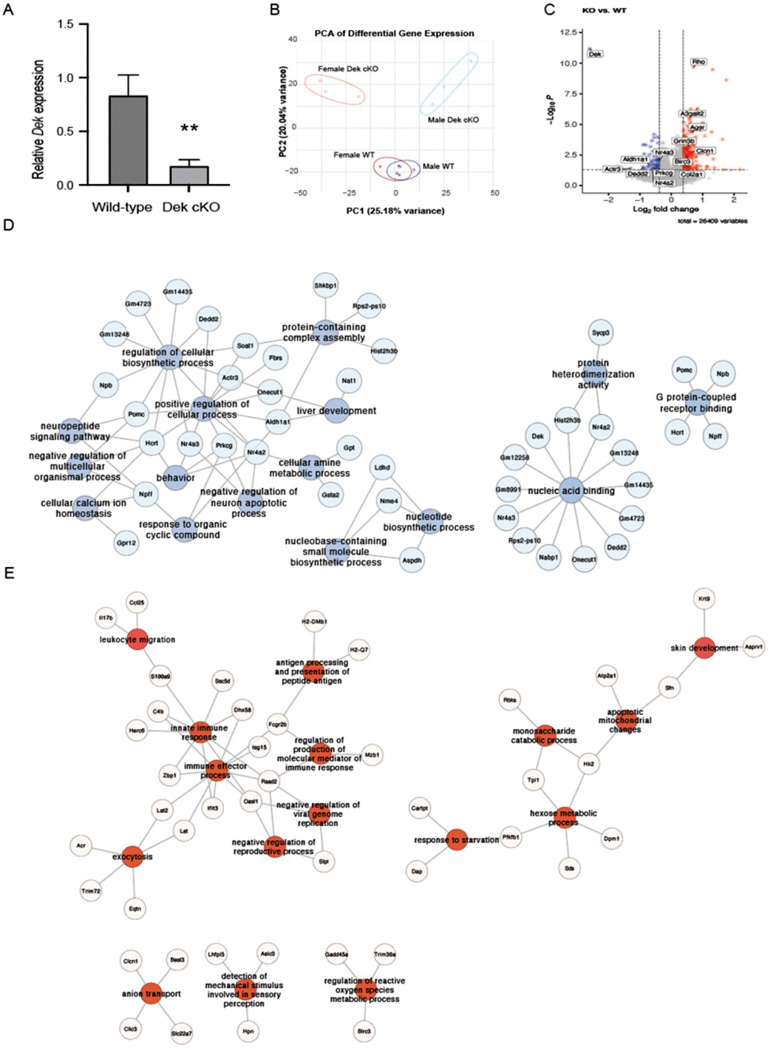
*Dek* loss leads to transcriptional deregulation of genes across multiple cellular processes. (A) *Dek* mRNA levels in the hippocampus of WT and *Dek* cKO mice using qRT-PCR shows loss of *Dek* expression in the *Dek* cKO mice. N=6 per group. (B) Principal component analysis (PCA) shows the separation of groups based on sex and genotype due to variation in the transcriptome using hippocampal mRNA. The ellipses represent 95% confidence intervals. N=3/genotype/sex. (C) The volcano plot depicts genes upregulated (red) and down-regulated (blue) in *Dek* cKO hippocampus compared with WT mice. (D-E) Differentially expressed genes from *Dek* cKO mice compared with WT mice were utilized to create cellular networks with Cytoscape, with cellular functions as central nodes and associated genes attached. Males and females were pooled for each genotype to find common deregulated genes. (D) Blue circles are down-regulated. Relevant down-regulated processes are nucleic acid binding, behavior, calcium homeostasis, and biosynthetic processes. and (E) Red circles are up-regulated processes. Up-regulated processes included immune response nodes, suggestive of neuro-inflammation.

The hippocampus is the primary brain region for long term and spatial memory. To investigate the molecular signature of the observed behavioral changes and hallmarks of *Dek* loss, we performed transcriptomic analysis of micro-dissected hippocampal tissue. First, we validated that *Dek* mRNA levels were significantly downregulated in the hippocampus of the *Dek* cKO mice compared with WT controls via RT-qPCR (Fig. 4A). We then performed bulk RNA-seq on the hippocampal tissue of three mice from each genotype and sex. Principal component analysis (PCA) confirmed the clustering of expression within each group. Minimal difference in gene expression between sexes of WT mice was confirmed by the overlap in male and female clusters (Fig. 4B). In contrast, male and female *Dek* cKO mice displayed greater separation from each other supporting greater variability, and from their respective WT groups supporting a sex difference in the transcriptomes of *Dek* cKO mice. We first compared *Dek* cKO mice with WT mice, regardless of sex. There were 251 genes upregulated, and 67 genes downregulated, between WT and *Dek* cKO mice (fold change +/− 1.3 and raw p value < 0.05); Fig. 4C). We performed gene ontology analysis and found these differentially expressed genes (DEGs) clustered in several nodes regarding cellular and molecular functions. Downregulated (blue) genes were associated with functions including calcium homeostasis, behavior, macromolecule biosynthesis, apoptotic processes, nucleic acid binding, and neuropeptide signaling (Fig. 4D). Upregulated genes (red) in the *Dek* cKO versus WT hippocampus largely clustered in immune response and hexose metabolic process pathways (Fig. 4E).

**Figure 5.**
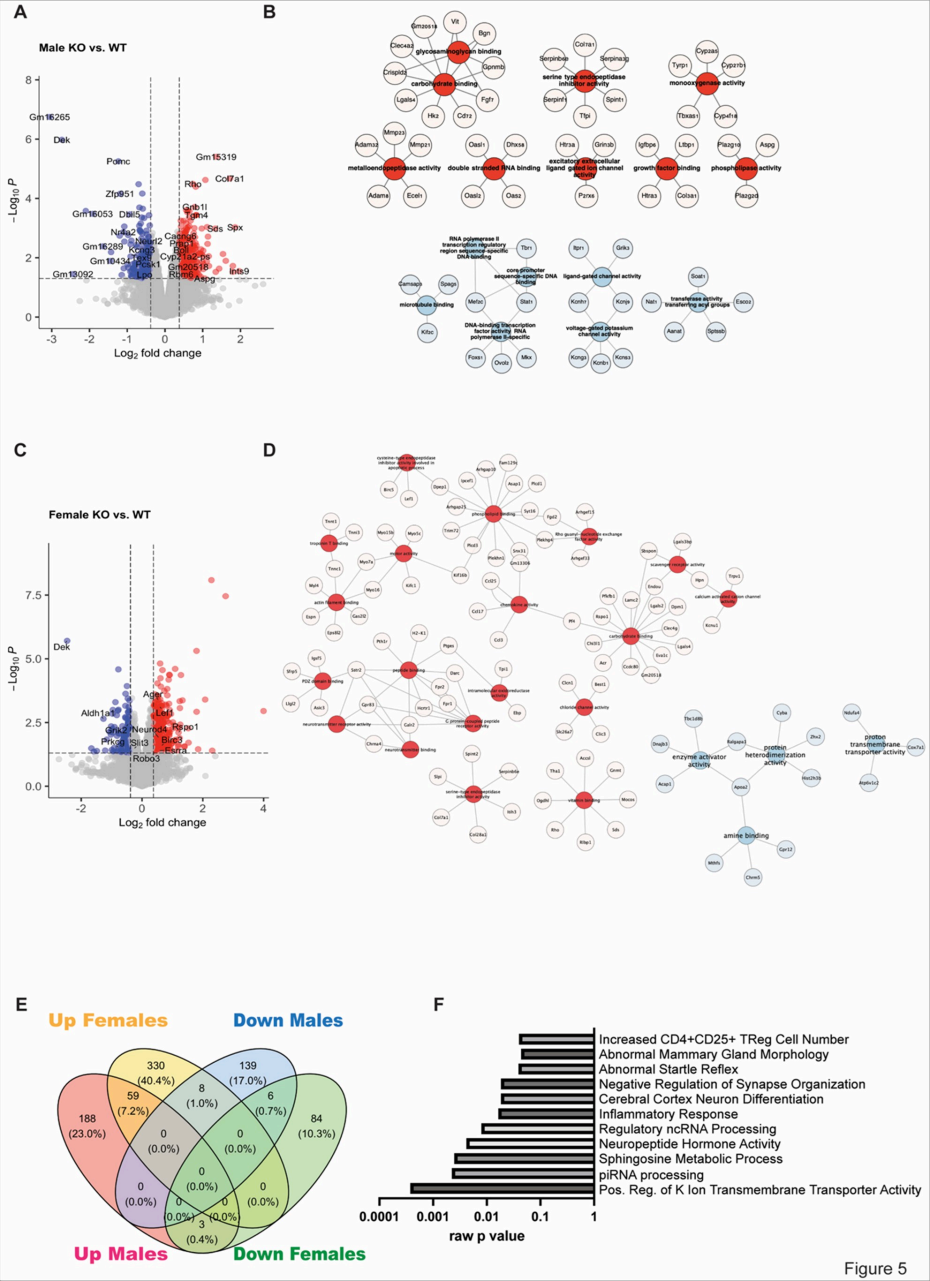
*Dek* loss leads to sex-specific transcriptomes in the mouse hippocampus. (A, C) The volcano plot depicts genes upregulated (red) and down-regulated (blue) in male (A) and female (C) *Dek* cKO hippocampus compared with WT mice. (B, D) Differentially expressed genes from *Dek* cKO mice compared with WT mice were utilized to create cellular networks with Cytoscape, with molecular functions as central nodes and associated genes attached. Molecular functions in males (B) were distinct from the molecular functions of DEGs in females (D). Blue circles are down-regulated and red are up-regulated processes. (E) A Venn diagram depicts the overlap of differentially expressed genes based on sex and *Dek* genotype. (F) The 76 shared DEGs from male and female *Dek* cKO mice were analyzed for gene ontologies using Enrichr (https://maayanlab.cloud/Enrichr/) N=3/sex/genotype

Next, we separated the sex and compared the transcriptomes of the two genotypes for each sex separately. Differentially expressed genes were identified based on a fold change +/− 1.3 and raw p values < 0.05. For males, *Dek* cKO hippocampal tissue had 250 up-regulated genes and 153 down-regulated genes when compared with WT tissue (Fig. 5A). GO-ELITE gene ontology pathway analysis determined that genes relevant for molecular functions of carbohydrate binding, serine-type endopeptidase inhibitor activity, double-strand RNA binding, ion channel activity, and more (red) while molecular functions associated with down-regulated genes included RNA polymerase II transcription, microtubule binding, and voltage-gated potassium channel activity (blue, Fig. 5B). Further gene set enrichment analysis (GSEA) identified down-regulation of dendrite morphogenesis genes (NES=−1.73, adj p = 0.00679), and upregulation of gene sets related to endopeptidase inhibitor activity (NES = 1.94, adj p = 4.96e-05), adaptive immune responses (NES = 1.63, adj p = 0.000307), ribosome RNA processing (NES = 1.74, adj p = 0.00192), regulation of inflammatory response (NES = 1.51, adj p = 0.033), and TNFR2 non-canonical NFkB pathway (NES = 1.8, adj p = 0.00856) (**Fig S3A**). For females, *Dek* cKO hippocampal tissue had 397 up-regulated genes and 93 down-regulated genes (Fig. 5C). GO-ELITE gene ontology pathway analysis determined that up-regulated genes were relevant for molecular functions of chemokine activity, phospholipid binding. Rho signaling, neurotransmitter receptor activity, vitamin binding, serine endopeptidase inhibitor activity, and both calcium and chloride channel activity (red). Down-regulated genes were related to molecular functions that included amine binding and enzyme activator activity (blue, Fig. 5D). GSEA identified very different processes when compared with GSEA analysis of the male DEGs. Female enriched gene sets included down-regulated reactome pathways such as PRC2 methylates histones and DNA (NES=−1.85 adj p = 0.0414), DNA double strand break response (NES=−1.83 adj p = 0.0414), and mitotic prophase (NES=−1.67 adj p = 0.0414). Enriched gene sets for upregulated genes in the hippocampus of *Dek* cKO females compared with WT females included the humoral immune response (NES = 1.71 adj p = 0.0042), collagen biosynthesis and modifying enzymes (NES = 1.88 adj p = 0.00879), and metal ion transmembrane transporter activity (NES = 1.53 adj p = 0.0369) (**Fig S3B**). Finally, we identified which genes were differentially expressed in both male and female Dek cKO compared with WT controls. Of the 403 DEGs in males and 490 DEGs in females, only 76 genes were shared (Fig. 5E). Six genes were down-regulated in both sexes while 59 genes were upregulated in both sexes. Interestingly, there were 11 genes that were regulated in different directions between the sexes. Eight genes were down-regulated in males but up-regulated in females, and these include *Zfp951, Nxph3, Ovol2, Tox, Sptssb, Tbr1, Sgpp2*, and *Vat1l.* Three genes (*Rbm5, Tmem267*, and *Orc1*) were up-regulated in male Dek cKO hippocampal tissue but down-regulated in female Dek cKO hippocampus (Fig. 5E). We did a gene ontology analysis using Enrichr to identify processes associated with the 76 common DEGs between sexes and identified neuron differentiation, abnormal startle reflex, inflammatory response, and neuropeptide hormone activity to be statistically significant gene ontologies (Fig. 5F).[36] When sexes were combined, we found 318 DEGs in *Dek* cKO mouse hippocampus compared with WT controls (Fig. 3G), but only 76 common DEGs when the sexes were divided (Fig. 5E). This may be due to the difference in statistical power for combining sex (n = 6/genotype) versus separating the sexes (n = 3/genotype) for analyses and the sex differences being lost in the combined analysis.

**Figure 6.**
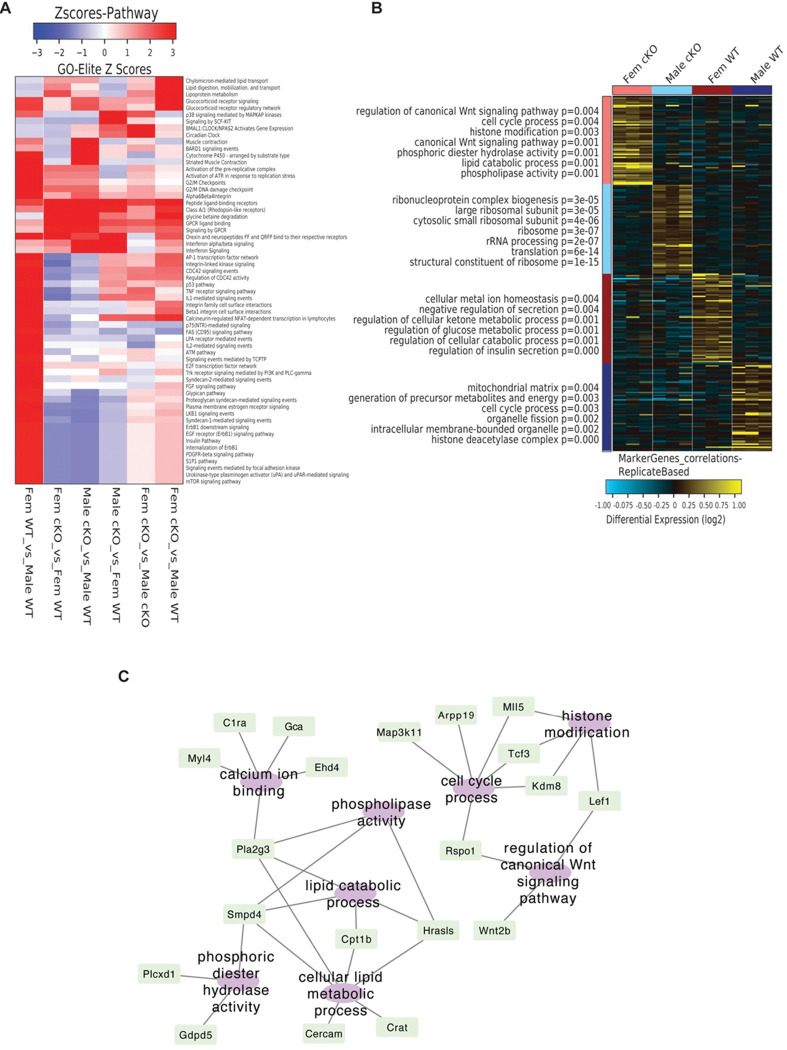
Identification of sex-specific signaling pathways in the hippocampus of WT mice, and identification of a unique transcriptional profile for female *Dek* cKO mice that highlights differences in dementia-relevant pathways. (A) A Heatmap depicts regulated pathways from the Pathway Commons database that are different in pairwise comparisons of all four sex X genotype groups, including sex differences in WT mice. Differentially expressed genes were identified using AltAnalyze with Kallisto and differentially expressed genes assess for pathway analysis using GO-Elite. The first column identifies sex-specific differences of the transcriptome from the hippocampal tissue of WT C57Bl/6 mice (Female WT vs. Male WT). Key upregulated pathways in female hippocampus from WT include DNA repair and stress pathways like ATM, ATR, and p53, in addition to mitogenic, pro-proliferative pathways like mTor, EGFR, FGF, and E2F transcription factor targets. In comparison, as shown in the second column, female *Dek* cKO mice compared with Dek WT females have down-regulation of these same pro-proliferative and DNA damage/stress pathways. This suggests that *Dek* cKO female mice have lost sex-specific differences and neuroprotective pathways. (B) Differentially expressed genes from female *Dek*cKO mice compared with female WT were utilized to create cellular networks with Cytoscape, with cellular functions as central nodes and associated genes attached. Blue circles are down-regulated and red are up-regulated processes. Relevant down-regulated processes are limited but include genes relevant to dendrites and synaptic membranes/synapses including the glutamate receptor *Grik2*and protein kinase C-gamma (*Prkcg*). Up-regulated processes included extracellular factors like paracrine signaling proteins, collagen, and basement membrane. Of interest, the sex-determining factor *Rspo1* is upregulated as well as several inflammation-relevant signaling factors like interleukins and pro-inflammatory *Ccl3*. The neurodegeneration and inflammation marker *Lrg1* is also upregulated in female *Dek* cKO mice. (C) Marker discovery analysis to identify genes that could uniquely identify each sex/genotype group was performed with MarkerFinder and AltAnalyze. A heatmap shows the differentially expressed genes that identify each group (yellow are up-regulated, and blue are down-regulated). Relevant gene ontologies and p-values are shown on the left. (D) Cytoscape network analysis was used to visualize the marker gene set for female *Dek* cKO mice, which identified Wnt signaling, lipid metabolism, and calcium ion binding as relevant pathways.

We then characterized the status of gene expression across molecular signaling pathways for each sex X genotype comparison using AltAnalyze and GO-Elite (supplementary data 2). Interestingly, this indicated a stark contrast between male and female WT mice and their hippocampal transcriptomes (Fig. 4A, first column). Compared with male mice, female WT mice had significantly upregulated pathways related to neurogenesis (E2F transcription factors), cellular stress (p38 and p53 signaling), DNA damage repair (ATM, ATR, and p53 pathways), and glucocorticoid receptor signaling while down-regulating circadian rhythm pathways. Notably, these sex differences in the transcriptome were largely lost, or diminished, when comparing female with male *Dek* cKO mice (Fig. 6A, second column from right). Finally, we performed marker analysis to identify a gene expression pattern that specifically identified female *Dek* cKO mice in comparison with all other groups (Fig. 6B-C). Pathways enriched in this female *Dek* cKO gene set included cell cycle processing, histone modification, phospholipid activity, canonical Wnt signaling pathway, and lipid metabolism. Overall, our results, both molecular and behavioral, indicate that *Dek* loss of function in female mice disrupts the expression of genes in pathways relevant to neuron vitality and function that may explain their sex-specific memory impairment.

## Discussion

The present study reveals that loss of DEK results in female-specific impairments in select cognitive domains, particularly those associated with executive functioning, including cognitive flexibility and pre-attentive sensory processing. These deficits were not observed in male *Dek* knockout (cKO) mice, nor were they evident in tasks assessing other forms of learning and memory, such as novel object recognition, conditioned fear memory, or spatial learning during the acquisition phase of the Morris Water Maze. This task-specific and sex-dependent pattern of impairment underscores the selective role of DEK in modulating cognitive processes beyond basic learning and memory abilities. While our previous work demonstrated sex differences in DEK expression in distinct brain regions associated with learning and memory, like the hippocampus [37], this study is the first to establish functional consequences of DEK loss in vivo. Notably, the behavioral deficits observed in female *Dek* cKO mice, particularly impairments in cognitive flexibility and pre-attentive processing, are broadly consistent with our previous findings in humans, where reduced DEK expression in the brain was associated with greater dementia severity in postmenopausal women with schizophrenia but not in men [32]. Although the human data were based on Clinical Dementia Rating (CDR) scores, which primarily reflect overall cognitive status rather than specific domains such as attention or executive function, the convergence of sex-specific effects across species is noteworthy. These findings suggest that DEK may have a conserved role in supporting cognitive resilience in females, especially in the context of aging or disease. The human *DEK* gene is located within a schizophrenia risk locus (6p23) [38], and previous studies have linked DEK to molecular features of neurodegeneration, including tau pathology [26–28]. However, this is the first demonstration that DEK loss selectively impairs cognitive performance in a sex- and task-dependent manner, providing new insight into mechanisms that may contribute to sex-specific vulnerability in neuropsychiatric and neurodegenerative disorders characterized by cognitive dysfunction.

Given that the most prominent behavioral deficit in female *Dek* cKO mice emerged during the reversal learning phase of the Morris Water Maze, we focused our initial transcriptomic analysis on the hippocampus to explore molecular pathways that may contribute to this sex-specific phenotype.[39] Although both female and male *Dek* cKO mice exhibited intact spatial learning during the acquisition phase, female knockouts showed selective impairments in reversal learning, suggesting a deficit in cognitive flexibility. This form of executive function enables individuals to adapt behavior in response to changing contingencies. While executive function is often attributed to the prefrontal cortex, increasing evidence shows that the hippocampus, and particularly the CA1 subregion, plays an important role in behavioral flexibility. Several rodent studies have demonstrated that CA1 is selectively engaged during reversal learning. For example, reversal learning in a Y-maze increases calcineurin activity in CA1 and CA3 but not in other hippocampal subregions, indicating a region-specific molecular response to cognitive adaptation.[40] In addition, long-term potentiation in CA1 is modulated during reversal phases of spatial tasks, underscoring the importance of synaptic plasticity in this subregion for updating previously learned information.[41] Although the dentate gyrus contributes to behavioral flexibility through adult neurogenesis, CA1 functions as a downstream integrator within the trisynaptic circuit and is critical for the flexible encoding and retrieval of memory.[42] Our previous work identified sex differences in DEK expression specifically within the CA1 region[11], which further supports the rationale for targeting the hippocampus in our molecular analysis. While not all the observed behavioral phenotypes, such as impaired pre-pulse inhibition, are directly mediated by the hippocampus, this region represents a biologically relevant starting point for identifying transcriptomic changes that may contribute to the sex- and task-specific effects of DEK loss on cognitive function.

Our transcriptomic data from the hippocampus suggests that elimination of *Dek* negatively impacts neuronal function. We discovered deregulated expression of genes involved with synaptic function, intercellular signaling, and calcium signaling, including down-regulation of calcium-responsive *Prkcg* (protein kinase C gamma) and of glutamate receptor *Grik2*. Glutamate excitotoxicity is a known molecular event associated with neurodegenerative diseases, which occurs through the disruption of glutamate-calcium homeostasis.[43] When glutamate transport is disrupted, intracellular calcium accumulates and glutamate levels increase in the synapse. Excess synaptic glutamate causes excess stimulation leading to both dysregulation of glutamate and electrochemical potential. Together, glutamate excitotoxicity and disrupted calcium homeostasis can induce Tau hyperphosphorylation and aggregation.[44] Protein kinase C is protective against glutamate excitotoxicity,[45] so the combined downregulation of *Prkcg*, glutamate receptor *Grik2*, and other calcium-associated proteins suggests that *Dek* loss may contribute to glutamate excitotoxicity and loss of calcium homeostasis. Interestingly, DEK over-expression has been linked to metabolic reprogramming and decreased extracellular and intracellular glutamate in a human epithelium model, which supports that DEK loss may result in excess glutamate and subsequent excitotoxicity.[46] Additional experiments are needed to confirm this causative inference for the sex specific differences in memory we observed.

Our data show functional and transcriptomic support for previous work by Rodriguez-Rodriguez *et al.* in which *Dek* loss in murine neurons causes epigenetic changes via histone post-translational modifications that impact gene transcription, and that *Dek* deficient neurons are hyperpolarized and had increased excitability while *in vivo Dek* loss led to Tau accumulation, microglia activity, and neuron loss.[28] Their reports of microglia activity with *Dek* loss, combined with our observation of upregulation of genes linked to immune system function, including pro-inflammatory factors like *IL-17b, Ccl3* and *Ccl17*, suggest that *Dek* deficiency contributes to neuroinflammation, a hallmark of neurodegenerative diseases. In our dataset, not only were several nucleic acid-binding genes deregulated in *Dek* cKO mice, but female *Dek* cKO mice specifically had differences in the expression of two genes involved in histone H3 methylation for epigenetic gene regulation: *Mll5/Kmt2e* (H3K4 site) and upregulation of lysine demethylase *Kdm8* (H3K36 site). Interestingly, Rodriguez-Rodriguez *et al* also reported the H3K36 locus to be epigenetically targeted by increased acetylation with Dek loss, which would require the site to be demethylated first by an enzyme like Kdm8. However, they did not test for sex differences in their model. Furthermore, in our sex-specific GSEA analyses, we identified loss of PRC2 complex activity, which creates the H3K27me3 epigenetic mark, in the list of female, but not male, gene ontologies for DEGs in *Dek* cKO mice. Recently, DEK loss in murine neural progenitor cells and mouse ES was found to decrease H3K27me3 levels and we recently reported that DEK loss correlated with less H3K27me3 in murine mammary epithelial cells.[47–49] These epigenetic mechanisms may be one explanation for the vastly different transcriptomes when analyzing the DEGs. Specifically, female *Dek* cKO mice had 71 down-regulated and 257 up-regulated DEGs that were not shared by males nor were in the list of DEGs when the sexes were combined. This suggests that these 328 genes are uniquely expressed in female *Dek* cKO but not male *Dek* cKO. Correspondingly, male Dek cKO had 280 DEGs that were unique to males (131 down- and 149 up-regulated genes) (**Fig S4**). These differences allowed the detection of unique marker gene sets that could be used to identify each sex and genotype group (Fig. 6).

Sex differences in cognitive function are, clinically, most commonly observed in AD, a neurodegenerative disease wherein 2/3 of patients diagnosed with AD are female.[2] The prevailing theory is that this disparity is because of a loss of estrogen, as it is a neuroprotective factor that decreases with age and has been linked to neurodegenerative diseases in post-menopausal women.[50] Interestingly, we did not identify any hormone receptors to be differentially expressed in our transcriptomic data. However, *DEK* is an ER-α target gene, and it functions as a chromatin remodeling protein essential for DNA replication, chromatin organization, and epigenetic modification.[9, 20, 35, 48, 51–53] Thus, DEK may be a key functional protein downstream of ER-a that mediates the neuroprotective functions of ovarian hormones like estrogens. Furthermore, *Dek* cKO females showed 2.3-fold upregulated *Rspo1* (R-spondin 1) expression in the hippocampus. R-spondin1 suppresses male differentiation during gonadogenesis and is essential for ovary formation by regulating Wnt signaling; indeed, its loss causes complete sex reversal in mice.[54] The role of RSPO1in the brain is largely unknown, but its role in sex determination may suggest that it impacts the function of sexually dimorphic tissues. Its upregulation in this context is intriguing and worthy of future investigation.

Our findings with sensorimotor gating deficits in female *Dek* cKO, as indexed by prepulse inhibition (PPI), underscore the involvement of a distributed neural network that includes the hippocampus, medial prefrontal cortex (mPFC), and other regions including the striatum.[55, 56] Impairments within this circuitry have been consistently associated with cognitive inflexibility, attentional dysfunction, and executive deficits in neuropsychiatric and neurodegenerative conditions, including schizophrenia and Alzheimer’s disease. These disorders are often characterized by early disruptions in pre-attentive processing and sensorimotor integration, which may precede broader cognitive decline.[57, 58] Notably, rodent models of both schizophrenia and Alzheimer’s disease also exhibit deficits in PPI, supporting the relevance of pre-attentive processing impairments across species and disease contexts.[59, 60] Future studies should aim to dissect the region-specific and circuit-level mechanisms through which *Dek* loss affects both cognitive and sensorimotor domains, with particular attention to understanding the biological basis of female susceptibility and relative male resilience. This line of investigation may offer important insights into sex-specific vulnerabilities across cognitive disorders, both with and without underlying neurodegeneration.

One limitation in our study is the use of the CMV-Cre promoter, leading to whole brain knockout for *Dek*, regardless of cell type or tissue. Tissue- and cell-specific loss of *Dek* in the brain are needed in future experiments to determine the cause of memory impairment. Furthermore, the mean age of the mice in each group was 6–8 months old, so some females in this group may not have yet reached ‘estropause’, similar to menopause, which occurs between 9–12 months of age.[61] Therefore, future work should consider an age effect in this model to account for both the effects of aging and the decrease in ovarian hormone levels in older rodents. Since *DEK* is highly expressed during fetal development in the human brain[29] we cannot rule out developmental defects in the *Dek* cKO mice with this model. Furthermore, we were limited in statistical power to compare the performance of males and females directly in behavioral assays using two-way ANOVA. Future work will need to expand upon the sample sizes here for confirmation of a sex effect. Another limitation is that, while these transcriptomic data are highly informative, they are still correlative and may not underlie the observed sex differences in behavior. Future mechanistic studies are needed to determine which of these genes and pathways, if any, are the causative factor(s) downstream of *Dek* loss that led to the sex-specific differences in cognitive and sensorimotor dysfunction reported here. An additional limitation of the present study is the sole focus on the hippocampus as a target region to identify the transcriptomic consequences of *Dek* loss. While our findings clearly demonstrate that females are particularly vulnerable to cognitive impairments in hippocampal-dependent tasks such as the Morris Water Maze, the female-biased deficits observed during the reversal phase of this task also implicate the medial prefrontal cortex (mPFC), a region critical for executive function and cognitive flexibility, particularly in reversal learning[62] This suggests that *Dek* loss in other brain areas beyond the hippocampus, including the mPFC, may contribute to the observed sex differences in cognitive performance.

To our knowledge, this is the first report describing the consequences of *Dek* deficiency in the brain on learning and memory. While previous studies have linked *DEK* loss to molecular and cellular features associated with dementia and neurodegeneration,[26–28] its functional impact on cognitive processes had not been assessed *in vivo*. Here, we show that *Dek* deficiency results in sex-specific effects on cognitive performance in mice, particularly in tasks that tap executive function and pre-attentive processing. These findings suggest that DEK may play a broader role in supporting cognitive function and brain health, and that *Dek* knockout mice could serve as a useful model for investigating sex-specific mechanisms underlying vulnerability to certain cognitive deficits.

## Materials and Methods

### Mouse Model

Handling of mice was performed with the approval of the Cincinnati Children’s Institutional Animal Care and Use Committee and approved under protocols 2020–0037, 2023–0043, and 2023–0022. Mice were housed in a controlled environment with a 12-h light/12-h dark cycle in specific pathogen-free housing, with free access to water and a standard irradiated chow diet (Lab Diet, Richmond, VA) and RO and UV sterilized water.

The *Dek* conditional knockout allele was generated using the CRISPR technology to introduce 5’ and 3’ loxP sites sequentially to flank exons 3 and 4 of this gene as described.[35] To test for phenotypes caused by *Dek* loss, *Dek*^*fl/fl*^ mice were bred to CMV-Cre mice on the C57Bl/6 background (Jackson Labs strain #006054). Once the deletion allele achieved germline transmission, the CMV-Cre transgene was eliminated from the colony by directed breeding and the strain was maintained as a constitutive KO line via mating of heterozygous (*Dek*^+/D^) males and females. For behavioral studies, the mean ages of the groups at the time of behavior testing were: female WT (27.9 weeks), female *Dek* cKO (28.5 weeks), male WT (23.7 weeks) and *Dek* cKO males (34.1 weeks). Mice chosen for transcriptomic analyses were from the same cohort as those tested in behavioral assays and were 6–12 months old at time of collection.

### Genotyping

Tail clips were digested with DirectPCR Lysis Reagent (Viagen Biotech) containing 0.6 mg/mL Proteinase K (Invitrogen) and protocol (need to read bottles/thermocycler). For PCR analysis 1 mL of DNA was added to JumpStart Taq Ready mix from Invitrogen (Carlsbad, CA, product # P2893) using the manufacturer’s specifications. *Dek* conditional knockout models were genotyped with the following primers

CMV-Cre:

Forward: GCGGTCTGGCAGTAAAAACTATC

Reverse: GTGAAACAGCATTGCTGTCACTT

*Dek* (detects endogenous and floxed alleles):

5’loxP Forward: AGTGAAATTACTGGTCTGTGAAG

5’loxP Reverse: CTGAGTGGAACAGCTCCTATAG

3’loxP Forward: AGATGCTTCACCTTAGAGCTG

3’loxP Reverse: TCAGTTTGGAGCAAATTTCATTTCC

*Dek* deletion allele:

Forward (5’loxP forward): AGTGAAATTACTGGTCTGTGAAG

Reverse (3’loxP reverse): TCAGTTTGGAGCAAATTTCATTTCC

Behavioral Testing: Mice were tested in the Animal Behavioral Shared Facility (RRID:SCR_022621) by personnel blinded to the genotype of the mice.

Locomotor Activity/ Open Field Test: To complete the open field test[63], mice were placed in SDI activity chambers (41 cm × 41 cm) for 60 min. Activity was monitored by the PAS system (San Diego Instruments, San Diego, CA) which records total beam breaks, ambulation (successive beam breaks) as well as center vs peripheral activity. Chambers were cleaned with Process NPD disinfectant (Steris Corp., St. Louis, MO), an EPA approved, non-toxic denaturing, anti-bacterial, and anti-viral agent, between the evaluation of every mouse.

Novel Object Recognition: Novel object recognition (NOR)[63] was initiated one day after the open field test and was divided into three phases: habituation, familiarization, and retention. AnyBox chambers were 40 cm × 40 cm made of clear acrylic (Stoelting Co., Wood Dale, IL) with a camera mounted above. Behavior was tracked with AnyMaze that scored time spent within 1 cm of an object. Day-1 mice were placed in the box with no objects for 10 min. On Day-2 mice were placed in the box with four identical objects placed in each corner with no room for the mouse to move behind the objects for 10 min. Day-3 was the same as Day-2 except with four different new identical objects. Day-4, Part-1 was with four new identical objects until the mouse accumulated 30 s of object exploration time up to a 10 min limit. Mice reaching the 10 min limit were re-tested 4–6 h later with four new objects; if it failed again, its data were not used. Day-4, Par-2 was 1 h later. For this (retention) 3 copies of the objects used during familiarization were placed in three corners and one new object was placed in the fourth corner. Mice were allowed to explore until they accumulated 30 s of object time. The dependent measure was time with the novel object.

Morris Water Maze: The Morris Water Maze (MWM) was initiated 4 days after NOR. The apparatus for the MWM consisted of a 150 cm diameter, 51 cm high, white tank with a 10 cm platform submerged 1–1.5 cm below water level. Water temperature was 21 ± 1°C. Data were collected using video tracking software (AnyMaze, Stoelting Company, Wood Dale IL). The procedure had four phases. For all phases mice were tested in rotation with ~ 10 min intertrial intervals.[63] Mice that failed to find the platform were removed and placed on the platform for 10 s.

Phase-1 was training with curtains surrounding the tank. A 10 cm platform with an orange ball (10 cm above the water) was attached via brass rod to the platform. Each trial was limited to 90 s or when the mouse found the platform. The start and platform locations were identical for each trial. Mice that failed to find the platform twice within the six trials were retested 4–6 h later. Mice that failed during re-testing were not included in subsequent phases.

Phase-2 was acquisition for 6 days and began the day after training. Curtains were opened to reveal distal cues positioned on the walls. A 10 cm submerged white platform was used. There are five days of learning and 1 memory (probe) trial on day 6. The mice underwent four trials each day with a 90 s limit per trial. There were 4 start positions (2 cardinal and 2 ordinal positions around the perimeter) used each day in a quasi-random order with the platform located in the same place on each trial. On day 6 the platform was removed, and a single 45 s trial was given with the mouse starting from a novel location. Acquisition provides data on distal cue learning, whereas the probe trial tests reference memory.

Phase-3, reversal, started the day after the acquisition probe trial. For this phase, curtains were opened, and a 7 cm platform was used with the platform in the opposite quadrant from acquisition. Again, there were five days of learning and a probe trial on day 6. These trials provide a test of cognitive flexibility.

Phase-4 was with a visible cue but this time the position of both the start and platform were changed on each trial (4 trials/day for 2 days). Curtains were closed around the tank to obscure distal cues.

Acoustic and Tactile Startle (ASR-TSR): Acoustic startle (ASR) and tactile startle responses (TSR) were initiated 4 days after the completion of the MWM and were assessed using a SR-LAB apparatus (San Diego Instruments, San Diego, CA).[64] Mice were placed in acrylic cylindrical holders mounted to an acrylic base with insulated legs with a piezoelectric accelerometer attached to the underside to detect movement. The platform was positioned inside a sound-attenuated cabinet, and the system was calibrated daily. The session began with 5 min of habituation with no stimulus. Startle was assessed for 3 days. Days 1 and 2 consisted of 100 trials of alternating blocks of 5 acoustic and 5 tactile trials with intertrial intervals of 20 s. The acoustic stimulus was a 20 ms, 120 dB (SPL) mixed frequency white noise sound burst with 1.5 ms rise time. The tactile stimulus was a 20 ms 60 psi air puff, delivered to the dorsal surface of the mouse through a tube inserted in the top of the animal holder. Prepulse inhibition (PPI) of startle was assayed on day 3. Each mouse received a 10 × 10 Latin square sequence of 5 trial types (200 trials), with prepulses of 0, 59-, 70-, 80-, or 93-dB sound bursts lasting 20 ms for both TSR and ASR with a 50 ms gap before onset of the pulse, either acoustic or tactile. The dependent measure was the maximum startle response (V_max_) measured in mV.

Conditioned freezing: Conditioned freezing was assayed 4 days after completion of the startle response tests and was assessed in San Diego Instruments apparatus ( San Diego, CA) in 25 cm × 25 cm test boxes as previously described.[63] Each acrylic chamber had speakers mounted on the underside of the lid and a grid floor connected to a foot shock generator. Habituation was on day 1, in which mice were placed in the apparatus for 10 min. On day 2, the conditioning phase, mice were placed in the chamber for an acclimation period of 6 min where there were no stimuli, followed by 6 conditioned stimulus (CS)-unconditioned stimulus (US) parings consisting of a tone and light with foot shock. The CS was an 85 dB, 2 kHz tone concurrent with the house light turning on. Each pairing consisted of the 30 s CS accompanied by foot shock (1.3 mA, though the gird floor) during the last 2 s. CS-US pairings were separated by 30 s intervals. On day 3 mice are returned to the chamber for 6 min with no stimuli and no shock and activity and freezing episodes recorded as an index of contextual memory. On day 4, cued memory was tested; mice were placed in a different chamber made black acrylic, hexagonal in shape with a solid floor. Mice were given 3 min of no stimuli followed by 3 min with light-tone without shock. This was followed by 10 extinction trials consisting of alternating light-tone intervals and of 30 s of no stimuli. Activity and freezing were analyzed.

### Bulk RNA-seq:

Tissue Collection: Mice were euthanized by isoflurane inhalation followed by decapitation within 6 weeks of the completion of the final behavior assay. Brains were microdissected to remove the hippocampus and stored in RNA-Later at −20 °C. Tissue was homogenized in 1 mL of trizol using a tissue disruptor (Qiagen). Tissues were incubated on ice for 5 min, 200 mL of chloroform added, and the solution vortexed for 15 s. Then, the solution was incubated on ice for 14 min and vortexed halfway through. Samples were centrifuged at 12000 rpm for 5 min at 4°C. The aqueous phase was transferred to a 1.5 mL tube, and the DNA phase was frozen at −80 °C. To precipitate RNA, 500 mL of 100% isopropanol alcohol was added to the aqueous phase. After inverting gently five times, the samples were incubated at room temperature for 10 min then centrifuged at 1200 × g for 15 min at 4 °C. The RNA pellet was washed in 1 mL of ice-cold 75% ethanol and then centrifuged for 15 min at 4 °C. The supernatant was removed and the pellet air dried in a sterile environment for up to 30 min, then reconstituted in RNase-free water.

Library preparation and sequencing: The RNA quality of each sample was checked with an Agilent 5300 Fragment Analyzer. All RNA samples had an RQN value between 8.1 and 9.6. As determined by InvitrogenTM QubitTM high-sensitivity spectrofluorometric measurement, 150 to 300 ng of total RNA was poly-A selected and reverse transcribed using Illumina’s TruSeq^®^ stranded mRNA library preparation kit. Each sample was fitted with one of 96 adapters containing a different eight base molecular barcode for high level multiplexing. After 15 cycles of PCR amplification, completed libraries were sequenced on an Illumina NovaSeqTM 6000 with a read depth of 20 million 100 bp paired-end reads per sample.

RNA-Seq Analysis: FASTQ files were generated for each sample across multiple sequencing lanes. Merged paired-end FASTQ files were created by concatenated all read1 FASTQ files by sample and all read2 FASTQ files by sample using the Linux cat command. Kallisto was used to generate transcript per million (TPM) gene-level estimates and analyzed using AltAnalyze (version 2.1.4.4; EnsMart100 database; GRCm38/mm10 mouse reference genome) open-source software.[65] Analyze was used for differential gene expression analysis, including GO-Elite pathway enrichment. Principal component analysis (PCA) was performed to visualize the variance in differential gene expression across samples. The differential gene expression data from AltAnalyze, represented as log2 fold-change values, was analyzed using R (v4.4.2). The prcomp() function in R with mean centering and unit variance scaling was applied. PC1 and PC2 were extracted and merged with sample metadata for visualization. Confidence ellipses representing 95% confidence intervals for each group were drawn: FemKO (pink), FemWT (red), MaleKO (light blue), and MaleWT (blue). Two parallel analyses were then performed: a two-group analysis comparing all *Dek* knockout (cKO) samples with all wild-type (WT) samples and a four-group analysis comparing *Dek* cKO vs. WT split into male and female. Putative marker genes were identified using MarkerFinder, which is run by default when performing differential gene expression analysis with AltAnalyze. Volcano plots were generated with all expressed genes using the Enhanced Volcano package in R (v4.2.1). Significant genes with an eBayes p-value ≤ 0.05 and at least a linear 1.3-fold change were indicated in red if upregulated and blue if downregulated. The data that support the findings of this study are available from the corresponding author (LMPV).

### RT-qPCR

1 mg of RNA, from above, was reverse transcribed using the Quantitect Kit (Qiagen) to make cDNA. SYBR Green PCR master mix was used to amplify cDNA using an ABI-7500 quantitative PCR machine (Applied Biosystems) and analyzed by the ΔΔCt method. Primer sequences were used at a concentration of 0.4 ng/μL each and the sequences include

mDek F: 5′-AACGTGGGTCAGTTCAGTGGC-3′

mDek R: 5′-TTCGCTGTTCACGCCTGACCT-3′

mActin F: 5′-GATATCGCTGCGCTGGTCGTC-3′

mActin R: 5′-ACCATCACACCCTGGTGCCTAG-3′

### Statistical Procedures

Behavioral data were analyzed for each sex separately using generalized linear mixed-effect models (SAS Proc Mixed, SAS Institute 9.4 TS, Cary, NC) [66] or unpaired two-tailed t-tests. The main effect was genotype, and the repeated measures were 5 min time points (open field), block of 5 trials (ASR/TSR), trial type (ASR/TSR with acoustic prepulses), trial (straight water channel, MWM cued training, and conditioned freezing), and day (MWM). Repeated measure factors were fit to autoregressive moving average or autoregressive [67] depending upon best fit of the corrected Akaike Information Criterion. Significant interactions were analyzed using the slice option in Proc Mixed since it controls the overall error term in the analyses. The estimation method for the covariance parameters was by the restricted maximum likelihood method. Kenward-Roger first order adjusted degrees of freedom were used; these can be fractional, and were calculated for Type III ANOVAs [68]. A folded F test was conducted prior to t-tests to determine of variances were equal and if not the Satterthwaite method was used. Significance was set at p ≤ 0.05.

## Supplementary Material

Supplementary Files

This is a list of supplementary files associated with this preprint. Click to download.

• supplementaryfigures92025.pdf

## Data Availability

All data are available in the main text or the supplementary materials. Mouse models and other unique resources are available upon request to the authors and may require a materials transfer agreement. RNA-Seq data is available by request to LMPV and through GEO. Access to the data on GEO is pending.

## Significance Statement

Women are more likely to experience cognitive decline with diseases like Alzheimer’s. One possible reason is the DEK protein, which is increased by ovarian hormones and expressed in the brain. Previous studies suggested that DEK loss can lead to features of Alzheimer’s disease and other age-related dementias, such as DNA damage, increased Tau protein accumulation, changes in neuron morphology, and cell death. In our study, female mice with *Dek* loss showed task-specific cognitive deficits and altered gene expression not evident in male mice with *Dek* loss. These data indicate that DEK is important for brain function, especially in females, and may help us understand why women might be more vulnerable to certain cognitive disorders.
